# HIV self-testing relative to the landscape of HIV testing in Ontario, Canada

**DOI:** 10.1177/09564624251324978

**Published:** 2025-03-02

**Authors:** Patrick O’Byrne, Maya Kesler, Lauren Orser, Michael Kwag, Brook Biggin, Christopher Draenos

**Affiliations:** 1 School of Nursing, 6363 University of Ottawa, Ottawa, ON, Canada; 2 Ontario HIV Treatment Network, 7938 University of Toronto, Toronto, ON, Canada; 3 7938 University of Toronto, Toronto, ON, Canada; 4 584047 Community Based Research Centre, Vancouver, BC, Canada

**Keywords:** screening, testing, Canada, self-test, HIV

## Abstract

**Background:**

HIV self-testing may help achieve the UNAIDS 95-95-95 targets because it has the potential to increase testing among equity-denied communities. In 2023, the Public Health Agency of Canada made a one-time $8 million investment into HIV self-testing. We sought to evaluate the outcomes of HIV self-testing, compared to serology in Ontario, Canada.

**Methods:**

We submitted data requests to all agencies involved HIV self-test distribution in Ontario, Canada for 2022-2023. We obtained matching data from the Public Health Ontario Laboratory. We then analyzed for unique test, unique tester, and positivity rate per testing modality.

**Results:**

During the analysis period, we found that the laboratory completed an average of 53,606 tests per month for an average number of 44,671 unique persons. For self-tests, there was an average of 1700 tests distributed per month to an average of 678 unique persons. The positivity rate for self-testing was 0.27%, compared to 0.1% for serology.

**Conclusions:**

Our results highlight that self-testing can play a role but will not, alone, achieve the UNAIDS 95-95-95 targets. In our jurisdiction, self-testing corresponded with a higher positivity rate but accounted for only a minority of new diagnoses. In short, HIV self-testing is a tool, but not the solution to the HIV epidemic.

## Introduction

The UNAIDS strategy for 2030 aims to have 95% of persons living with HIV diagnosed, 95% of those who are diagnosed linked to care, and 95% of those in care achieving suppressed viral loads.^1^ These targets are referred to as the 95-95-95 goals^1^ – and they serve both to improve the health and wellbeing of persons living with HIV (via engagement in HIV care) and to decrease HIV transmission at the population level (via undetectable viral loads). Notably, the entry point to the 95-95-95 cascade is testing: to know one needs HIV treatment, one must undergo testing. Obtaining testing, however, can be fraught with barriers,^2^ including the costs of getting to or of accessing care, geographical distances to testing locations, and hours of operation or a lack of available appointments. Other factors also impede access to testing, such as patients or providers not perceiving any risk for HIV acquisition and stigma or discrimination at the point-of-care.

HIV self-testing kits have been proposed as one way to overcome some of these barriers because these devices can provide a private and convenient method to take an HIV test, possibly facilitating earlier diagnoses and timelier treatment.^3^ People do not need to travel to and wait in clinics and interact with healthcare professionals. Instead, these kits involve collecting blood from a fingerstick and they offer results in minutes. Saliva samples can also be used for self-testing; however, this approach is not yet approved in Canada (location of this study). Research moreover suggests that self-testing can boost testing rates within equity-denied communities and mitigate stigma around HIV testing.^4–6^

Building on these potential benefits, in 2023, the Public Health Agency of Canada (PHAC) made a one-time $8 million investment into HIV self-testing^7^ to make a fingerstick blood HIV self-test (as the only test licensed in Canada) readily and freely available. The money from PHAC was used to purchase and distribute such tests without a cost to participants at in-person and outreach events and through online mailout programs. This distribution of HIV self-tests occurred through two PHAC-funded programs, plus through GetaKit, which was funded by the Ontario HIV Treatment Network. Through these programs, individuals could obtain free HIV self-tests that they could use themselves and/or distribute within their networks. The PHAC-funded projects were available to anyone who requested testing, whereas GetaKit was risk-based and only provided testing to people who had a clinical indication for HIV testing based on Ontario guidelines.^8^

Due to this large investment and the potential for self-testing to influence the HIV epidemic in Canada, we set out to understand the landscape of HIV self-testing relative to serology. To do this, we reviewed HIV testing by self-testing and serology in Ontario, which is the most populous province in Canada with approximately 18 million residents. Ontario also has centralized HIV laboratory testing and an HIV self-testing program (i.e., GetaKit), which collected data that matched the laboratory system. For our analysis, we focused on the numbers of each test being performed and their positivity rates. We then focused more specifically on the GetaKit data to compare it to the laboratory outcomes. Our main question was, *What was the impact of such a large investment in self-testing on HIV testing volumes in Ontario?* A secondary question was, *What were the outcomes associated with self-testing compared to serology?*

## Methods

### HIV testing in Ontario

Historically, screening modalities for HIV in Ontario^9^ have included serology (using a fourth generation antigen-antibody combination test) and point-of-care testing (using a third generation rapid antibody test). The turn-around time for HIV serology in Ontario is 2–6 days^9^ and can be obtained by visiting almost any clinical setting (e.g., walk-in clinic, STI clinic, primary care). These clinical settings offer access to testing by walk-in and/or appointment. Point-of-care HIV testing in Ontario, meanwhile, is only available in designated sites via walk-in and appointments. For performance, this device has an estimated sensitivity of 99.9% and specificity of 99.5%, and provides results in 1–5 minutes.^10^ All such HIV testing is free for persons seeking testing and it can be completed nominally or anonymously (in designated sites province-wide).

HIV serology is processed almost single-source by the Public Health Ontario Laboratory.^9^ HIV point-of-care results are submitted to this laboratory as well, with the expectation (but not requirement) that persons with reactive HIV point-of-care tests have a blood sample submitted to confirm the positive test result. To conform a diagnosis, HIV self-tests similarly require confirmatory serologic testing that is submitted to the Public Health Ontario Laboratory for analysis.

For epidemiologic surveillance in Ontario, only positive serology results and point-of-care tests with positive confirmatory serology results are counted as diagnoses. Unconfirmed positive point-of-care and self-tests are not considered new diagnoses. The HIV requisition in Ontario,^9^ moreover, has a section for clinicians to indicate “reason for testing”, which includes options such as routine testing, acute or chronic symptoms, sexual assault, prenatal, use of HIV pre- or post-exposure prophylaxis, and positive HIV self-test. This information is routinely not submitted on the HIV requisition but can help contextual testing when it is available.

In November 2020, self-testing became a new modality after Health Canada licensed the bioLytical INSTI® HIV self-test,^11^ which is a rapid vertical flow through test that uses 50mcL of fingerstick blood to produce qualitative results for the presence of antibodies to HIV. Its performance is considered equivalent to the existing third generation HIV point-of-care test,^11^ albeit with higher invalid rates, likely related to its use by untrained operators.^12^ Free access to these tests occurred through the PHAC-funded program and the Ontario-funded initiative. People could also purchase these tests from the manufacturer or pharmacies at a cost of about $60 each.

### Data collection and analysis

Extended requests were submitted to PHAC and GetaKit for self-testing data for 2022-2023. We extracted data from the Public Health Ontario Laboratory system for HIV serology done in the same period. As is typical for analyses of HIV testing, we excluded prenatal screening so that we focused our laboratory-based screening on persons who were undergoing testing due to risk factors, not due to antenatal follow-up. For analysis, we clustered testing to monthly intervals and calculated the average overall number of tests that were performed (serology, inclusive of point-of-care tests) or distributed (self-tests); we repeated this monthly average for unique persons undergoing testing by both modalities. We calculated monthly averages, as it allowed us to control for differences in time periods between the datasets we obtained. For our sub-analysis on test outcomes, we calculated test positivity rates for each testing modality by dividing the number of reported positive results by the total number of tests performed, total numbers of unique persons who obtained testing, and total number of results reported back. Due to data availability, this sub-analysis occurred only for the laboratory-based testing and the GetaKit dataset.

### Ethics

Surveillance of HIV testing and diagnosis is standard public health practice in Ontario and is exempt from research ethics board approval. The University of Ottawa Research Ethics Board approved the GetaKit study.

## Results

**Figure 1.**
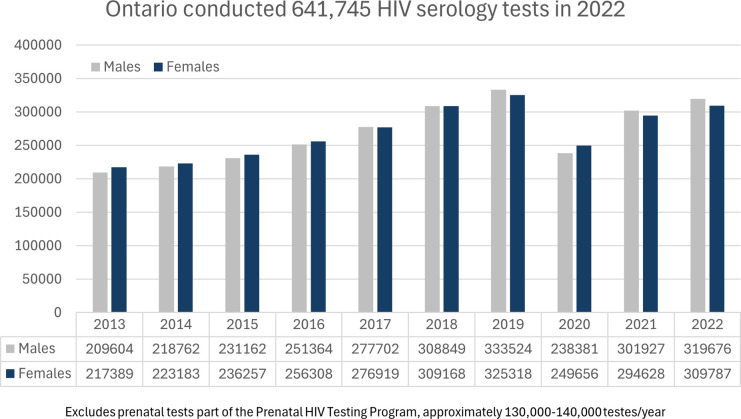
HIV testing by serology in 2022 in Ontario.

**Figure 2.**
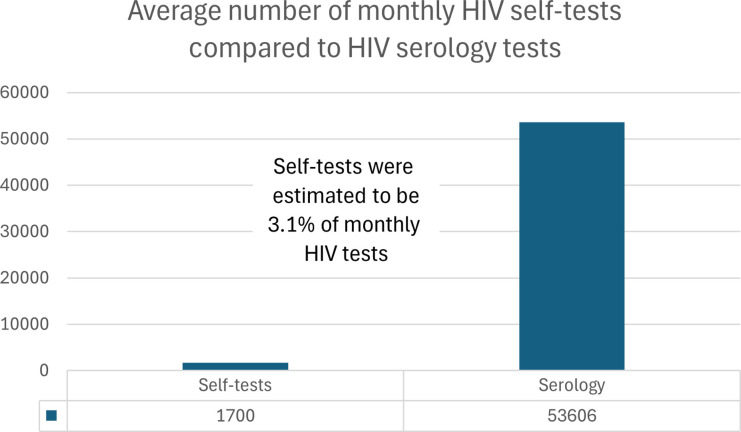
Average number of tests per month.

**Figure 3.**
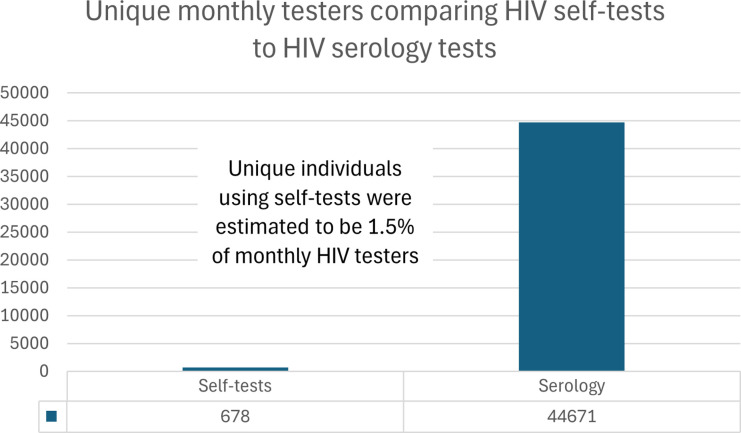
Average number of unique persons per month.

In 2022, excluding prenatal tests, the Public Health Ontario Laboratory system completed 641,745 HIV serology and point-of-care tests, with slightly higher numbers among males (319,676) compared to females (309,787). (Figure 1). This testing volume in 2022 equated to an average of 53,606 HIV blood tests being processed per month in Ontario and an average number of 44,671 unique persons being tested for HIV in Ontario per month. (Figures 2 and 3). The overall HIV testing volume in 2022 was roughly equivalent to that in 2018-19, but higher than during the height of the COVID-19 pandemic (2020-21).

Data on the HIV self-tests were obtained for November 2022 to June 2023. No data were available for direct-from-manufacturer or pharmacy purchases. Through GetaKit, 2570 HIV self-tests were distributed to 1944 unique persons. For the PHAC-funded programs, in Ontario, 9594 HIV self-tests were distributed to 2967 unique persons. Combined, there were 12,164 HIV self-tests distributed to 4911 unique persons, which yielded averages of 1700 self-tests distributed per month during the analytic period and 678 people given self-tests per month during this same period. Self-testing thus accounted for 3.1% of HIV tests performed in Ontario per month and 1.5% of unique persons who underwent HIV testing per month. (Figures 2 and 3).

Test positivity was available from the Public Health Ontario Laboratory for serology and point-of-care and from GetaKit. Diagnostic data were not available for the PHAC-funded projects. For GetaKit, 52% (*n* = 1334/2570) of orders had results reported back to the program; 7 of these were positive, generating positivity rates of 0.27% for all ordered tests, 0.36% for unique testers, and 0.52% for reported results. Two of the 7 people who reported positive results to GetaKit indicated that this was their first time doing HIV testing; all 7 positive self-test results reported to GetaKit were confirmed as true positive results through serology. This reporting rate from GetaKit means we had results for 11% (1334/12,164) of the HIV self-tests that were distributed from November 2022 to June 2023. For the Public Health Ontario Laboratory, 623 people had a first-time HIV diagnosis in Ontario in 2022, with positivity rates of 0.1% for serology and 0.2% for point-of-care. Test positivity rates for the HIV self-tests were thus nearly 3-times that of serology and 1.5- to 2-times that of point-of-care. Seven positive test results over 8 months equates to approximately 1 positive test per month from GetaKit, compared to approximately 52 positive test results from HIV serology per month. Self-testing through GetaKit thus accounted for 1.9% of new HIV diagnoses in Ontario per month during the analytic period. Lastly, 26 HIV serology tests were conducted in 2022 in Ontario with a clinician indicating that a positive HIV self-test was the reason for testing. No data were available on what proportion of these self-tests were confirmed as true positive test results. Data were also not available regarding what proportion of these 26 persons were first-time testers or if any arose from either the PHAC-funded projects or from GetaKit.

## Discussion

In this paper, we reviewed HIV self-testing relative to the full landscape of HIV testing in Ontario. We used data from the Public Health Ontario Laboratory and from the PHAC-funded initiatives and the Ontario-based GetaKit project, focusing on data from 2022 for the laboratory system and from November 2022 to June 2023 for self-testing. We also completed a sub-analysis of the GetaKit dataset regarding self-test positivity rates, comparing these data to those from laboratory-based testing in Ontario. We found that, per month in Ontario, HIV self-testing accounted for 3.1% of monthly HIV tests and 1.5% of monthly testers. We also found a positivity rate of 0.27% for self-testing through GetaKit, compared to 0.2% for point-of-care and 0.1% for serology. Diagnoses from GetaKit furthermore accounted for approximately 2% of HIV diagnoses in Ontario during our period of analysis. These results raise a few points for discussion.

First, our data highlight that self-testing accounted for a very low volume of overall HIV testing and an even smaller proportion of the overall number of people who completed HIV testing in Ontario during the period we analyzed. Self-testing also accounted for only a fraction of those who were diagnosed with HIV during our analytic period. We do not interpret these findings to signal that self-testing is ineffective. Instead, we take these data to suggest that self-testing is a new and likely successful way to link some – even if only a few – people to testing and treatment. Giving people access to different options for testing appears to be a successful strategy, especially considering that 2% of HIV diagnoses in Ontario arose from one of the self-testing initiatives (GetaKit alone).

We also do not take these data to suggest that self-testing should replace serology. We believe that our findings reinforce the utility of serology as the gold standard for HIV screening, diagnosis, and linkage-to-care. HIV self-testing is simply a new tool to add to, and improve upon, existing efforts and interventions. We posit that this is likely especially true for persons who are unable or unwilling to access serological testing, due to cost, access issues, or negative experiences in healthcare. Such findings and interpretations of our data align with the existing literature, which shows that self-testing can supplement, but not replace, traditional means of HIV testing, diagnosis, and linkage-to-care. With such caveats, self-testing initiatives in Ontario can be considered successful.

Second, while based on only a small segment of the total number of HIV self-tests that were distributed in Ontario during the period we reviewed, the positivity rates we identified from the GetaKit data are worth commenting on. For one, our data differed from published meta-analyses^13,14^ of HIV self-testing, which did not identify significant differences in overall positivity rates between self-administered and traditional HIV testing modalities, such as clinic-based serologic testing. Indeed, our review of the GetaKit data found higher positivity rates compared to serology, further highlighting a potentially important (albeit likely niche) role for this device. One reason why this finding might have emerged in our review is because GetaKit did not provide testing to the entire population, but focused testing on persons in the groups who have higher rates of HIV in Ontario (i.e., men who have sex with men [MSM], members of Indigenous communities, persons of African, Caribbean, or Black Ethnicities, and persons who inject drugs). Interestingly, from the extant literature, sub-analyses^14^ involving HIV self-testing among MSM similarly demonstrated HIV positivity rates almost double that of serologic testing, suggesting that this testing device might be most useful when distributed within higher prevalence networks. We take this finding to highlight the importance of targeted risk-based distribution of HIV self-tests.

Alternatively, differences in positivity rates for HIV self-testing compared to serology may relate to project design between observational cohort studies and randomized controlled trials. A recently published randomized controlled trial^15^ in England and Wales among 10,111 MSM participants, who were randomized in a 3:2 ratio to receive an HIV self-test or serology, found that “the offer of a single free HIV self-test did not lead to an increased rate of new HIV diagnoses”^15,p.1^, but that “the offer of a free HIV self-testing kit [simply] resulted in high HIV testing rates”^15,p.1^. The outcome of Roger et al.’s work^15^ was thus more testing – but without corresponding increases in diagnosis. This leads us to posit that the higher positivity rates that have been observed with self-testing projects involving MSM (which we also identified in our GetaKit dataset) might relate to an otherwise overarching inaccessibility of HIV serology (as described above). Stated differently, we surmise that the higher positivity rates we observed might not relate to self-testing at all and leaves us wondering if the diagnostic outcomes of serology for MSM may in fact be non-inferior to self-testing if serology were made more accessible. Further research is required on this point.

Third, we think our results highlight two important next steps in achieving the UNAIDS 95-95-95 targets.^1^ First, we need to better understand the motivations of persons who used HIV self-testing, focusing on those who were diagnosed with HIV and especially on those who were diagnosed with HIV and reported that this was their first time doing HIV testing. Understanding why self-testing was adopted by this group could improve future deployments of self-tests in ways that maximize individual and population health outcomes – and which limit needless resource consumption related to the indiscriminate distribution of these devices. Simultaneously, as we have speculated above, we must also make serology more accessible.^2,16^ To accomplish this, we must explore novel testing strategies,^16,17^ such as online testing platforms like GetCheckedOnline or the more recent GetaKit STI platform expansion, outreach efforts, faster diagnostic turn-around-times, and shorter window periods, etc. Ongoing efforts to decrease racism, sexism, homophobia, transphobia, and all other forms of discrimination in healthcare must also occur. Second, we cannot assume that obtaining a self-test naturally translates into meaningful outcomes. We only obtained 52% of results reported back for GetaKit (only data available to us), and our previous work on linkage-to-care^18^ showed that only 2 of 16 people who reported positive HIV self-test results to GetaKit had initiated next steps to obtain HIV care before our nursing team reached out to them. Published meta-analyses^14,15^ similarly found low rates of linkage-to-care for persons who do HIV self-testing.

Taken as a whole, our data and the published literature make it clear that we cannot assume that HIV self-testing will yield engagement in HIV care without any additional resources or interventions. We need to proactively and intentionally establish clear and readily accessible linkage-to-care pathways^19,20^ for persons who do self-testing. Otherwise, we may diagnose some people, but we will do this at the risk of leaving them without access to medication (thus not achieving viral suppression) and without the social supports they might require. The outcome could then be improvements in the first of the UNAIDS 95-95-95 targets (i.e., diagnosis), but no changes related to the metrics that actually improve the quality of life for persons living with HIV (i.e., linkage to care and the ability to achieve viral suppression and immune system restoration). In this light, we must expand access to testing but do so in ways that support access to care.

### Limitations

Our data must be interpreted considering certain limitations. First, we used restricted and different timelines for data analysis. Changes in subsequent years might have altered our findings. However, testing data are relatively stable and no additional funding announcements were made. Monthly testing averages therefore should have been consistent if we had expanded or changed the analytic period. Second, we did not have positive test results for all self-test programs. The positivity rates may have been higher if people did the self-test and did not report positive results, or they may be lower if – like with COVID self-tests – people hoarded these devices but did not use them. There are no data to inform us on this point. Third, secondary self-test distribution may have also yielded additional testing and/or diagnoses, but we were unable to link this back to any testing program. This could mean that the true outcomes of these HIV self-testing initiatives were more impactful than what we identified. Fourth, there are no data about directly purchased HIV self-tests, as we were unable to obtain these from the manufacturer. We therefore do not know how many people obtained HIV self-testing in this manner. However, in the context of the PHAC- and Ontario-funded initiatives which delivered free kits, we do not anticipate much uptake through direct-to-consumer purchases at $60 each. The number of such tests was likely very small in comparison and would not have changed our outcomes.

## Conclusion

As we move toward the 2030 target year for the UNAIDS 95-95-95 goals, HIV self-testing has become a new tool in Canada to help achieve these aims. To determine the potential impact of self-testing regarding uptake and diagnostic outcomes, we reviewed data from a PHAC-funded initiative and from the Ontario-based GetaKit. We compared these data to standard-of-care HIV serology in Ontario. Our findings highlighted that, although the volume of self-tests was proportionally low relative to serology, self-testing did yield a higher positivity rate and did reach at least 2 people who had never previously undergone HIV testing. It also accounted for at least 2% of HIV diagnoses in Ontario during the study period we analyzed.

We took these findings to mean that self-testing could be an important addition to the HIV diagnostic landscape, provided (1) that it is better understood (in relation to why some first-time testers opted for this approach), and (2) that the findings of these future inquiries are used to maximize targeted self-test kit delivery and linkage-to-care outcomes. We also took our findings to signal that access to HIV serology needs amelioration. Our concern is that, without improvements in access to serology, we will only promote older technologies (i.e., the third generation HIV self-test) and will do so without offering supports and services to people who test and who test positive. HIV self-testing is thus a viable strategy to complement serology but is not a panacea to achieve the UNAIDS 95-95-95 targets.^1^ If our goal is to achieve the UNAIDS 95-95-95 targets, a more comprehensive approach to HIV prevention, diagnosis, and care is needed – and one which positions the person who undergoes testing not as a faceless nameless person who needs diagnosis, but as a person to support in HIV prevention, diagnosis, and management. When we re-humanize HIV testing in this way, perhaps then we will be able to help 95% of persons living with HIV know their status and ensure that 100% of these people receive the care they deserve.

## References

[1] UNAIDS . Fast-track - ending the AIDS epidemic by 2030. https://www.unaids.org/en/resources/documents/2014/JC2686_WAD2014report (2014), Accessed 23 Oct 2023 from.

[2] Laprise C Bolster-Foucault C . Understanding barriers and facilitators to HIV testing in Canada from 2009–2019: a systematic mixed studies review. Can Commun Dis Rep 2021; 47(2): 105–125. DOI: 10.14745/ccdr.v47i02a03. 33746619 PMC7968477

[3] UNAIDS A . Short technical update on self-testing for HIV. https://www.unaids.org/sites/default/files/media_asset/JC2603_self-testing_en_0.pdf (2013), Accessed 23 Oct 2024.

[4] Centers for Disease Prevention & Control . Issue brief: the role of HIV self-testing in *Ending the HIV epidemic*. https://www.cdc.gov/hiv/policies/data/self-testing-issue-brief.html#:∼:text=It_can_also_be_easier,potential_transportation_and_geographic_barriers (2024), Accessed 24 Oct 2024.

[5] O’Byrne P McCready L Tigert J , et al. First-time testers in the GetaKit study: conceptualizing new paths to care for gbMSM. Health Promot Int 2023; 38(2): 1–7. 10.1093/heapro/daad029 PMC10132581 37099678

[6] O’Byrne P Musten A McCready L , et al. HIV self-testing enabled access to testing for Black persons: the GetaKit study. Res Nurs Health 2023; 46(2): 235–241. 10.1002/nur.22293 36573589

[7] Public Health Agency of Canada [PHAC] . News release: government of Canada is making HIV testing more accessible across Canada. Government of Canada is making HIV testing more accessible across Canada - Canada. ca, 2022.

[8] Ministry of Health, Government of Ontario . Ontario guidelines for providers offering HIV testing. https://hivtestingontario.ca/ontario-guidelines-for-providers-offering-hiv-testing/ (2024, Accessed on 25 Jan 2025).

[9] Public Health Ontario . HIV – diagnostic serology. https://www.publichealthontario.ca/en/Laboratory-Services/Test-Information-Index/HIV-Diagnostic-Serology (2024), Accessed 24 Oct 2024.

[10] Singh A Lee B Fenton J , et al. The INSTI HIV-1/HIV-2 antibody test: a review. Expert Opin Med Diagn 2013; 7(3): 299–308. 23480561 10.1517/17530059.2013.774370

[11] Government of Canada . Regulatory decision summary - INSTI HIV SELF TEST - health Canada. https://hpr-rps.hres.ca/reg-content/regulatory-decision-summary-medical-device-detail.php?linkID=RDS11033 (2020), Accessed 25 Oct 2024.

[12] O’Byrne P Musten A Orser L , et al. Invalid results in the GetaKit study in Ottawa: A real-world observation of the INSTI® HIV self-test among persons at risk for HIV. J Assoc Nurses AIDS Care 2022; 33(5): 567–573. 35472138 10.1097/JNC.0000000000000335 PMC9398507

[13] Jamil MS Eshun-Wilson I Witzel TC , et al. Examining the effects of HIV self-testing compared to standard HIV testing services in the general population: a systematic review and meta-analysis. EClinicalMedicine 2021; 38: 100991. DOI: 10.1016/j.eclinm.2021.100991. 34278282 PMC8271120

[14] Witzel TC Eshun-Wilson I Jamil MS , et al. Comparing the effects of HIV self-testing to standard HIV testing for key populations: a systematic review and meta-analysis. BMC Med 2020; 18: 381. DOI: 10.1186/s12916-020-01835-z. 33267890 PMC7713313

[15] Rodger AJ McCabe L Phillips AN , et al. Free HIV self-test for identification and linkage to care of previously undetected HIV infection in men who have sex with men in England and Wales (SELPHI): an open-label, internet-based, randomised controlled trial. Lancet HIV 2022; 9(12): e838–e847. DOI: 10.1016/S2352-3018(22)00266-1. 36460023 PMC7614584

[16] Hamilton A Shin S Taggart T , et al. HIV testing barriers and intervention strategies among men, transgender women, female sex workers and incarcerated persons in the Caribbean: a systematic review. Sex Transm Infect 2020; 96: 189–196. 31506346 10.1136/sextrans-2018-053932 PMC7062576

[17] Sundararajan R Ponticiello M Nansera D , et al. Interventions to increase HIV testing uptake in global settings. Curr HIV AIDS Rep 2022; 19(3): 184–193. DOI: 10.1007/s11904-022-00602-4. 35441985 PMC9110462

[18] O'Byrne P Kroch A Orser L , et al. Targeted HIV self-testing identifies persons with undiagnosed HIV and active engagement links them to care: the GetaKit study. AIDS Behav 2024; 28(6): 2015–2022. DOI: 10.1007/s10461-024-04302-5. 38526637 PMC11161533

[19] Levison JH Del Cueto P Mendoza JV , et al. Systematic review and meta-analysis of linkage to HIV care interventions in the United States, Canada, and Ukraine (2010-2021). AIDS Behav 2023; 27(12): 4070–4083. DOI: 10.1007/s10461-023-04121-0. 37523048 PMC10598185

[20] Dave S Peter T Fogarty C , et al. Which community-based HIV initiatives are effective in achieving UNAIDS 90-90-90 targets? A systematic review and meta-analysis of evidence (2007-2018). PLoS One 2019; 14(7): e0219826. DOI: 10.1371/journal.pone.0219826. 31314764 PMC6636761

